# When resilience backfires: the counterintuitive effect of employee resilience in high-tech surveillance environments

**DOI:** 10.3389/fpsyg.2026.1790846

**Published:** 2026-03-19

**Authors:** Jialu Chu, Hong Chu

**Affiliations:** 1 School of Business, Macau University of Science and Technology, Macau, China; 2 Tianjin College, University of Science and Technology Beijing, Tianjin, China; 3 University of Science and Technology Beijing, Beijing, China

**Keywords:** AI-awareness, artificial intelligence (AI) surveillance, autonomy, mental health, psychological distress, psychological resilience

## Abstract

The fast adoption of Artificial Intelligence (AI) in workplaces is a serious paradox: optimization tools can negatively affect the mental health of employees. The research paper examines the two psychological mechanisms that connect AI exposure with wellbeing among managers in the manufacturing industry in China. Based on the Stimulus-Organism-Response (S-O-R) framework, we discuss how AI-based surveillance and AI-awareness have different effects on psychological wellbeing mediated by the parallel mediators of perceived autonomy and psychological distress, and how the relationships are moderated by employee resilience. The structural equation modeling was performed on data of a three-wave time-lagged survey of 482 managers. Findings affirm that surveillance negatively impacts psychological wellbeing by lowering autonomy and enhancing distress, and awareness does so through the same mechanisms. As opposed to the buffering hypothesis, resilience increased the positive relationship between surveillance and distress, and undermined the protective role of awareness on distress. These results indicate that resilience is a two-sided sword in high-control AI settings, i.e., the issue of its consistent value, as well as the necessity of context-specific application of AI and support systems.

## 1 Introduction

The introduction of artificial intelligence (AI) into the working processes of an organization is no longer an imaginary future but a hallmark of the modern workplace. Starting with manufacturing floors and moving to service sectors, AI-based systems are bound to bring unprecedented productivity, operational accuracy, and strategic decision-making (Raisch and Krakowski, 2021; Schmid and Dowling, 2020). However, such an efficiency quest frequently neglects a very serious human paradox: the same technologies that are supposed to streamline performance may also destroy the psychological composition of the workforce (Trenerry et al., 2021). This two-sidedness highlights the necessity to redefine digital transformation as not only a technological change, but as a socio-technical transformation with direct implications to the morale, motivation, and mental health of employees (Kumar et al., 2024).

One of the key, but psychologically less understood manifestations of this development is the emergence of AI-based surveillance—the automatic, uninterrupted and often non-transparent tracking of employee actions, performance, and even moods (Mettler, 2024). Leaving the physical surveillance behind, such datafication of work forms an atmosphere of constant algorithmic assessment, and the ethical issues of privacy and data sovereignty are already well-documented (De Cremer and Narayanan, 2023). The psychological corollaries, the less clear ones, are, however, the less clear. Such surveillance may become a powerful stressor, which can lead to the development of feelings of anxiety, powerlessness, and a sense of control that has been eroded (Babu and Joseph, 2024; Cram et al., 2022). The concept of AI-awareness, i.e., an employee being aware of what AI systems can and cannot do in their work, is in stark contrast to it. The concept of transparency and knowledge, which the Explainable AI (XAI) movement promotes, is believed to address the fear factor, foster trust, and rebuild the sense of agency (Shin, 2021; Tariq et al., 2021). Therefore, the identical technological environment has two opposing stimuli, one of control and uncertainty, the other of clarity and possible empowerment.

These two opposing stimuli pose an underlying empirical question: in what psychological mechanisms do they influence employee psychological wellbeing, and does individual resilience change these mechanisms? The two research questions that will guide the study are as follows: RQ1: What are the specific mediating roles of perceived autonomy and psychological distress in the associations between AI-based surveillance/AI-awareness and psychological wellbeing? RQ2: What is the moderating effect of employee resilience on these pivotal relationships?

To make sense of how these stimuli result in employee psychological wellbeing, it is important to unravel the psychology behind the process. Based on Self-Determination Theory, perceived autonomy, which is the feeling of volition and choice in the workplace, is a key motivation nutrient and a psychological health nutrient (Ryan and Deci, 2017). By introducing an algorithmic control, AI-based surveillance threatens to frustrate this need, whereas AI-awareness can help to fulfill it by demystifying technology and making it possible to interact with it (Jia et al., 2024). Similarly, psychologically, transactional stress, psychological distress- anxiety and strain- is a direct affective route by which threatening environmental demands damage mental health (Lazarus and Folkman, 1984). The concurrent analysis of these two directions is the crucial gap in the literature that would give a more comprehensive view of the psychological impact of AI.

Moreover, the workers do not remain inactive receivers of technological stimuli. Differences in individuals are certainly known to influence their appraisal and coping reactions. The universal buffer is traditionally theorized as employee resilience, which is generally regarded as the ability to adjust to and survive in the face of adversity (Aguiar-Quintana et al., 2021). However, this supposition should be questioned. On high-pressure settings, opaque monitoring, resilience can be used as a two-sided sword. High-achievement striving, a trait that is often described as resilient, may have an individual who perceives constant surveillance as a challenge to overcome with better performance, which may internalize more pressure and develop greater distress, a phenomenon that the recent scholarship started to suggest (Glavin et al., 2024; Mahdiani and Ungar, 2021). The question of how resilience mediates the outcomes of both negative and positive AI factors is an open and complicated issue.

This investigation is enriched with the cultural and industrial background. The manufacturing industry of China, which is a giant in the process of the fast modernization based on AI, is a unique environment. This environment is high power distance and collectivist and can heighten the psychological experience of top-down surveillance as well as develop specific, group-oriented coping strategies (Farh et al., 2007; Hofstede Insights, 2023). The investigation of the interaction between AI stimuli, psychological processes, and individual resilience in this particular setting challenges the extrapolability of Western-centric models and examines the key cultural boundary conditions.

To address these gaps, the current research formulates and evaluates a composite model that will be informed by the S-O-R framework (Russell and Mehrabian, 1977). We locate AI-based surveillance and AI-awareness as a stimulus of the environment (S), perceived autonomy and psychological distress as internal organismic state (O), and psychological wellbeing as the final response (R). More importantly, we include employee resilience as a mediator of stimulus-organism relationship. The two research questions that lead to this investigation are as follows: What are the unique mediating effects of perceived autonomy and psychological distress in the associations between AI-based surveillance/AI-awareness and psychological wellbeing? So what moderating effect does employee resilience have on these critical relationships? Through answering these questions, this study will add a finer, mechanism-heavy insight into how to manage the human costs and benefits of integrating AI into the workplace.

## 2 Conceptual framework and hypotheses development

### 2.1 Theoretical foundation: extending the S-O-R framework

In order to untangle the psychological processes that workplace AI technologies influence the psychological wellbeing of employees, the present study is based on the S-O-R paradigm (Russell and Mehrabian, 1977). The framework assumes that environmental stimuli (S) trigger internal cognitive and affective states in an individual (O), which in turn influence his/her responses (R). Its usefulness is rooted in the fact that it does not focus on direct effects but reveals the underlying processes of the human psyche that are activated by new environmental factors, including the ones brought by algorithmic management (Tang et al., 2022).

AI-based surveillance and AI-awareness are two separate, yet crucially important, environmental stimuli (S) in our model in the contemporary workplace. They are two aspects of the AI-enabled environment, one of which is focused on control and observation, and the other on transparency and understanding. Two central processes, perceived autonomy, which is the cognitive-evaluative channel that is associated with self-determination and control, and psychological distress, which is the affective-emotional channel that is associated with strain and anxiety, capture the internal organismic states (O) (Liu and Miao, 2022). The last response (R) is psychological wellbeing, which is defined as a positive mental functioning and satisfaction at work (Hussain et al., 2022).

Based upon this, we also define the constitutive nature of these technological stimuli in the S-O-R paradigm. The conceptualization of AI-based surveillance as an imposed environmental stressor is an imposition-top-down, and in most cases, opaque system of control that imposes external demands on employees. Conversely, AI-awareness is understood as an attainable mental asset-knowledge and wisdom offered by the organization or pursued by the individual to make sense of the AI environment. This is an important difference that points to the fact that the same technological infrastructure can be evaluated as a source of strain or as a source of clarity thus creating the context of their divergent effects on the organismic states (O).

We augment the traditional S-O-R framework with the employee resilience as an important boundary condition. The resilience is a conceptualized individual difference variable that is capable of modifying the strength or even the direction of the relationship between technological stimuli (S) and internal states (O). This anthropocentric form of integration recognizes that employees are not inactive consumers of technological impositions but active participants whose individual resources are essential in determining their psychological and behavioral response to AI (Dong and Yan, 2022).

The choice of self-determination and suffering as fundamental processes is theoretically conscious. Autonomy is one of the cornerstone psychological nutrients based on Self-Determination Theory (SDT); its satisfaction results in psychological wellbeing, whereas its frustration results in ill-being (Ryan and Deci, 2017). Simultaneously, in terms of transactional stress (Lazarus and Folkman, 1984), psychological distress reflects the adverse affective consequence of the environmental demands, e.g., opaque AI-based surveillance, when perceived as a threat or surpassing the coping means. Our model, by placing the two constructs in parallel, provides an opportunity to capture both the motivational (through autonomy) and affective (through distress) pathway, providing a more detailed explanation of how AI influences employee psychological wellbeing.

### 2.2 AI-based surveillance: Undermining autonomy and elevating distress

#### 2.2.1 AI-based surveillance and perceived autonomy

The AI-based surveillance that is usually continuous and algorithmically motivated is fundamentally based on the logic of control and optimization. In employee perspective, such widespread and often invisible surveillance can have a devastating effect on a sense of perceived autonomy the sense of volition and choice in the work one does and how they do it (Deady et al., 2024). Algorithms management, in contrast to conventional human supervision, is not negotiable and pre-established metrics, giving employees little opportunity to act at their discretion or make situational decisions (De Cremer and Narayanan, 2023). Staff members can then feel that the system is dictating their behavior instead of it being their professional judgment, and they are reduced to mere cogs in the computational machine. This feeling of powerlessness is further compounded by the nature of the lack of transparency in data collection and evaluation (Cram et al., 2022; Lammi, 2021). This is supported by recent studies on algorithmic control, which indicate that it may result in a sense of agency loss and a feeling of being constrained (Wood et al., 2019). We therefore hypothesize:

*H1*: AI-based surveillance is negatively associated with perceived autonomy.

#### 2.2.2 AI-based surveillance and psychological distress

The feeling of constant surveillance by some inexplicable algorithmic mechanism is a serious occupational stressor (Olawade et al., 2024). The black box aspect of most AI systems can create chronic uncertainty and hyper-vigilance as employees might expect unjust assessments or penal consequences on the basis of poorly comprehended standards (Babu and Joseph, 2024). This anticipatory stress together with a feeling of lack of control is psychologically draining and may lead to emotional burnout. Additionally, the detached quality of algorithmic supervision may destroy interpersonal trust and increase a sense of isolation, which are effective causes of distress (Mettler, 2024). New empirical data keeps growing in the association of electronic performance monitoring with the rise of anxiety and strain (Ravid et al., 2022). Thus, we propose:

*H2*: AI-based surveillance is positively associated with psychological distress.

### 2.3 AI-Awareness: fostering autonomy and mitigating distress

#### 2.3.1 AI-awareness and perceived autonomy

Psychological empowerment can be developed in stark contrast to surveillance, which is the awareness of the role, capabilities, and limitations of AI systems in the workplace, and the concept of AI-awareness. The better employees understand the way AI tools operate and how decisions are made, possibly with the help of Explainable AI (XAI) principles, the more effectively they will be able to interact with those systems, anticipate the results, and feel a sense of agency (Shin, 2021). This information de-mystifies the technology, making it a predictable threat, and an asset to be used, thus enhancing a sense of competence and control. Awareness would allow employees to adjust their work methods and possibly use AI to make better decisions themselves, which would strengthen a sense of autonomy (Jia et al., 2024). Technology transparency research confirms its contribution to user autonomy and participation (Shin, 2021). Hence, we hypothesize:

*H3:* AI-awareness is positively associated with perceived autonomy.

#### 2.3.2 AI-awareness and psychological distress

The presence of ambiguity and lack of knowledge about AI is a major cause of anxiety, which inspires fears of job loss, fair treatment, and technological obsolescence. AI-awareness serves as the key remedy as it decreases these uncertainties and creates predictability (Mahbooba et al., 2021). Being transparent about the purpose of AI and the logic behind its work will help to establish trust in the technology and the organization itself, which will reduce the views of AI as an all-powerful and scary power. Awareness will help reduce psychological distress by offering a coherent cognitive framework to interact with AI and more adaptively assimilate it into work routines (Tong et al., 2021). The recent research indicates that the lack of stress among employees is linked to the sense of clarity regarding the goals and boundaries of AI (Shin, 2021).. Therefore, we posit:

*H4:* AI-awareness is negatively associated with psychological distress.

### 2.4 Mediating pathways: autonomy and distress

Grounded in SDT and stress theory, we position perceived autonomy and psychological distress as the core mechanisms linking AI factors to psychological wellbeing.

Satisfaction of the need for autonomy is essential for fostering high-quality motivation, engagement, and ultimately, psychological wellbeing (Deady et al., 2024). When employees feel a genuine sense of choice and ownership, they experience greater vitality and satisfaction. Conversely, environments that thwart this need—as AI-based surveillance might—undermine mental health. Thus:

*H5:* Perceived autonomy is positively associated with psychological wellbeing.

Chronic psychological distress depletes cognitive and emotional resources, impairing effective functioning and the capacity to experience positive states (Hamouche, 2023). It is a well-established antecedent to burnout and diminished psychological wellbeing. The stress generated by AI-related appraisals is therefore hypothesized to directly corrode overall mental health:

*H6:* Psychological distress is negatively associated with psychological wellbeing.

### 2.5 The contingent role of employee resilience

Employee resilience—the capacity to adapt positively to adversity—is expected to critically shape how AI-related stimuli are interpreted and processed. Conventional wisdom casts resilience as a buffer, attenuating negative impacts. Following this, one might expect resilient individuals to better maintain autonomy and manage distress under surveillance, perhaps by reframing challenges or employing proactive coping strategies (Glavin et al., 2024). Similarly, resilience could amplify the benefits of awareness, as resilient employees may more actively seek and utilize knowledge for mastery. Yet, the question of whether these buffering predictions will be true or not depends crucially on how resilient employees perceive AI-related demands, which we can answer with the help of the challenge-hindrance framework and the regulatory focus theory.

However, the stress-buffering perspective is based on a very strict premise: that every employee would rate AI-based surveillance as a threat. This is challenged in the challenge-hindrance framework (Cavanaugh et al., 2000) which subdivides stressors into challenge stressors, which are demand but can be overcome conditions that elicit approach-oriented motivation, and hindrance stressors, which hinder goals and elicit avoidance. The identical environmental situation can be rated in different ways according to individual traits and we believe that employee resilience is the most important variable that changes this rating.

The mechanism is offered by regulatory focus theory (Higgins, 2012). The individuals who are highly resilient and have high achievement-striving and personal agency (Smith et al., 2008; Lin and Yan, 2023) work in a promotion regulatory orientation, which involves viewing challenging environments as mastery arenas. Faced with AI-based surveillance, such employees are, in theory, more likely to evaluate continuous algorithmic surveillance not as an aspect that should be avoided but as an aspect of performance that should be overcome. This challenge appraisal has a peculiar price: instead of staying apart of the evaluative metrics, the strong employee internalizes it, building self-established norms that are equal to or even more than what the system expects. What occurs is a kind of pressure generated on oneself which only increases, but does not reduce distress- the pressure amplification effect. This is further exacerbated by perfectionistic tendencies, which may co-occur with resilience (Mahdiani and Ungar, 2021), making every cycle of evaluation a referendum of personal adequacy.

The identical rationale produces a converse prediction of AI-awareness. The low-resilience employees who work in the prevention focus depend on the clarity awareness as an outer scaffold to deal with uncertainty. Employees who are highly resilient, and who have strong internal regulatory resources, get decreasing marginal returns to this informational support- a resource substitution effect that is also in line with the Conservation of Resources theory (Hobfoll, 1989). Consciousness thus alleviates distress among high-resilience employees than among low-resilience employees.

These arguments generate two sets of theoretically competing predictions, formalized as hypotheses below.

*H7a:* Employee resilience weakens the negative relationship between AI-based surveillance and perceived autonomy.

*H7b:* Employee resilience weakens the positive relationship between AI-based surveillance and psychological distress.

*H7c:* Employee resilience strengthens the positive relationship between AI-awareness and perceived autonomy.

*H7d:* Employee resilience strengthens the negative relationship between AI-awareness and psychological distress.

**FIGURE 1:**
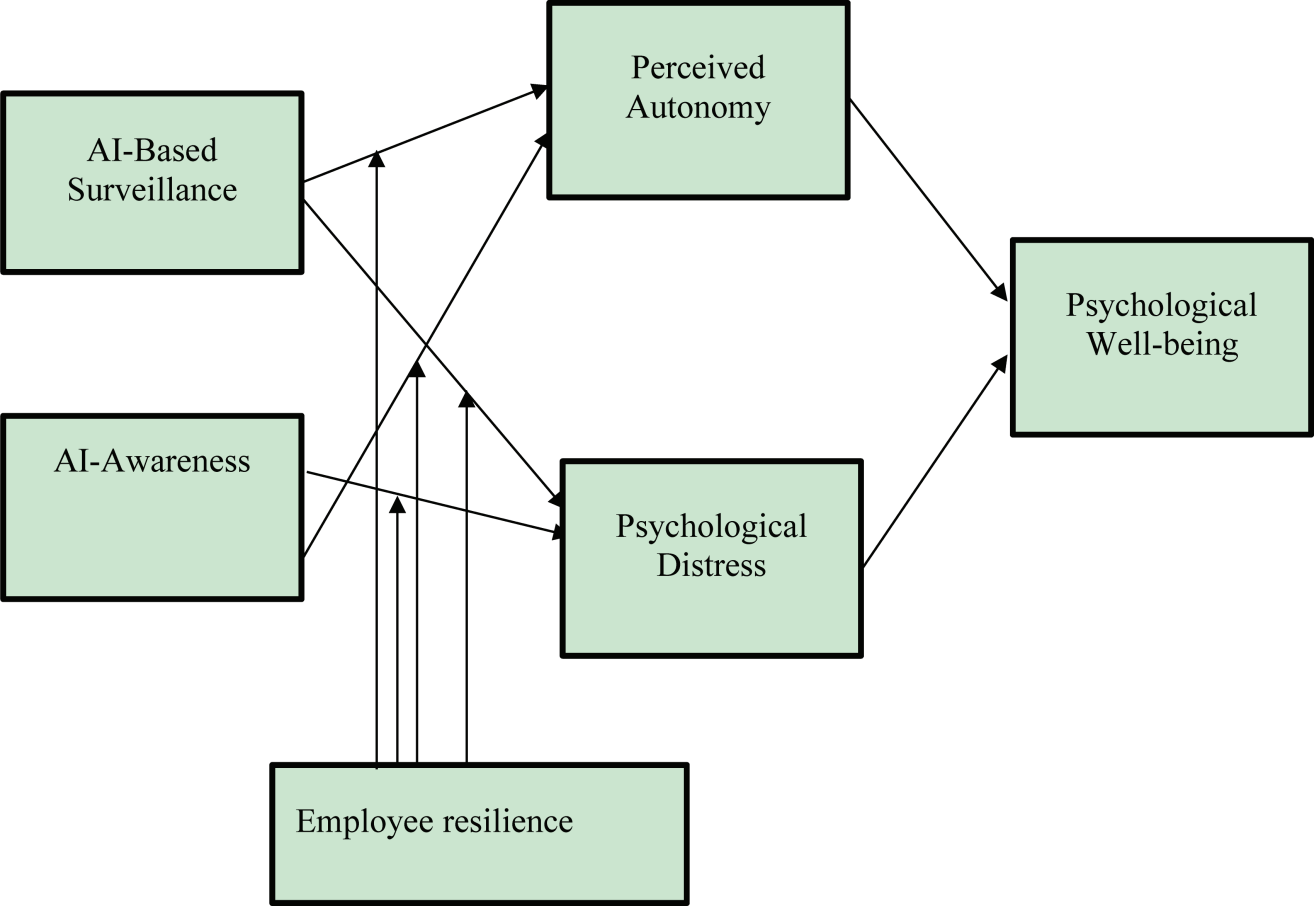
The conceptual research model. Solid arrows represent hypothesized direct and mediating paths. Dashed arrows signify the hypothesized moderating effects of employee resilience. For detailed hypothesis development, see sections 2.2–2.5.

The complete set of these hypothesized relationships is integrated within our conceptual model, as visually summarized in Figure 1.

## 3 Methodology

This study employed a multi-wave survey design to investigate the psychological pathways linking workplace AI exposure to managerial psychological wellbeing in China. The research context, sampling strategy, and procedural safeguards were carefully selected to bolster causal inference and mitigate methodological artifacts—a crucial consideration given the perceptual nature of the core constructs (Podsakoff et al., 2012). The following sections detail the methodological architecture.

### 3.1 Research design and data collection procedure

The research was centered on the manufacturing industry of China, which is an area that is experiencing massive AI adoption in performance management, predictive maintenance, among other activities (Chiu et al., 2021). The selection of managerial workers was not random; they are the ones who tend to be exposed to and often implement AI-based performance systems, and thus, they are both at the crossroads of AI application and the psychology of teams (Lee and See, 2004). The purposive sampling was used to recruit participants who were medium- to large-sized manufacturing enterprises in three major industrial provinces, including Guangdong, Jiangsu and Zhejiang in cooperation with corporate human resources departments.

The operationalization of the term manager was broad enough to reflect the entire range of managerial experience with AI-based performance systems: it includes frontline supervisors (team leaders with direct control over production workers; 33.8%), middle managers (department or section managers; 45.4%), and senior managers (plant or general managers; 20.8%). This hierarchical range was planned: The intensity and autonomy of AI-based surveillance is likely to vary at different levels, and its capture allows one to investigate whether the effects can be generalized across the managerial hierarchy. To be eligible, one had to have a minimum of 1 year of managerial experience and must have direct and daily exposure to AI-based performance systems.

The sample covers four major manufacturing sub-sectors of electronics and semiconductor (29.5%), automotive and machinery (24.5%), consumer goods and textiles (20.1%), and chemical and materials (15.4%), with the rest being represented by other manufacturing situations (10.6%). The participating organizations were of medium size (300–999 employees; 39.2), large (1,000–4,999; 42.1), and very large size (5,000+; 18.7). Regarding ownership, the sample consists of state-owned enterprises (28.6%), domestic firms that are privately owned (40.7%), and foreign or joint-venture enterprises (30.7) that give the variation in organizational governance that could influence the implementation and experience of AI-based surveillance. In 18.5% of cases, between 1 and 3 years and over 3 years, AI systems had been operating at participating organizations < 1 year, 1–3 years and more than 3 years, respectively, a range that represents organizations at meaningfully different levels of AI integration maturity. The sample profile is compiled in Table 1.

**TABLE 1:** Sample profile.

Characteristic	n	%
Managerial level		
Frontline supervisor (team leader)	163	33.8
Middle management (department/section manager)	219	45.4
Senior management (plant/general manager)	100	20.8
Industry sub-sector		
Electronics & semiconductor manufacturing	142	29.5
Automotive and machinery manufacturing	118	24.5
Consumer goods and textile manufacturing	97	20.1
Chemical and materials manufacturing	74	15.4
Other manufacturing	51	10.6
Organization size (number of employees)		
Medium (300–999)	189	39.2
Large (1,000–4,999)	203	42.1
Very large (5,000+)	90	18.7
Ownership structure		
State-owned enterprise (SOE)	138	28.6
Private domestic enterprise	196	40.7
Foreign / joint-venture enterprise	148	30.7
AI implementation duration at organization		
< 1 Year	89	18.5
1–3 Years	201	41.7
More than 3 years	192	39.8
Province of recruitment		
Guangdong	196	40.7
Jiangsu	163	33.8
Zhejiang	123	25.5
Gender		
Male	257	53.4
Female	225	46.6
Age		
25–29 Years	67	13.9
30–39 Years	175	36.4
40–49 Years	158	32.8
50 Years and above	82	17.0
Managerial tenure		
1–5 Years	61	12.7
6–10 Years	182	37.8
More than 10 years	239	49.6

The 3 months gap was chosen both theoretically and empirically. In theory, SDT studies suggest that the need frustration that persists over weeks to months to yield consistent changes in psychological wellbeing necessitates weeks to months, as opposed to the acute changes that can be observed in days (Gagné and Deci, 2005; Van den Broeck et al., 2016). Empirically, previous research of electronic monitoring and employee strain has used a range of 6 weeks to 6 months, 3 months being an intermediate at which cross-lagged effects on affective outcomes are reliably observed (Ravid et al., 2022; Cheng et al., 2024). The 3-month interval was chosen as a period deemed sufficient for the psychological impacts of AI exposure (e.g., on autonomy or distress) to manifest in more stable psychological wellbeing assessments, while minimizing attrition.

Data collection proceeded as follows:

Time 1 (T1): Participants reported their perceptions of AI-based surveillance and AI-awareness.
Time 2 (T2), 3 months post-T1: Measures of the hypothesized mediators, perceived autonomy and psychological distress, were administered.
Time 3 (T3), 3 months post-T2: Participants completed scales assessing psychological wellbeing and the moderating variable, employee resilience.

To ensure accessibility and compliance in diverse industrial settings, paper-based questionnaires were distributed on-site via HR offices. Each wave included a unique identifier to allow longitudinal matching while preserving anonymity. Rigorous follow-up protocols were used to verify continued employment and willingness to participate. Out of the 800 managers contacted, 511 responses were obtained in all the waves (63.88%). Among them, 29 cases (5.68) were dropped through listwise deletion because of incomplete or internally inconsistent responses, which left 482 participants (effective response rate: 60.25%). Comparisons of excluded and retained cases on all T1 variables (AI-based surveillance, AI-awareness, age, gender, tenure, and managerial level) showed no statistically significant differences (all *p*s > 0.05; all Cohen d 0.10) indicating that missingness was random. To test the robustness, we re-estimated the structural model with full information maximum likelihood (FIML); none of the paths changed substantially, which is evidence of the sufficiency of the listwise solution.

### 3.2 Measures

All constructs were measured using established multi-item scales, adapted where necessary for the context of AI and managerial work. Responses were captured on a five-point Likert scale (1 = strongly disagree, 5 = strongly agree), except for psychological distress, which used a frequency scale (1 = never, 5 = very often). All adapted scales underwent a rigorous translation and back-translation process, and their psychometric properties were validated within our sample.

AI-Based Surveillance was assessed with a 3-item scale adapted from recent work on electronic monitoring (Glavin et al., 2024). A sample item is: “AI-based technologies are used to track my work activities and progress.”

AI-Awareness was measured using a 4-item scale adapted from Brougham and Haar (2018), focusing on understanding AI’s role and boundaries. An example item is: “I have a clear understanding of how the AI systems at work make decisions that affect my job.”

Perceived Autonomy was gauged with a 3-item subscale from the Work-Related Basic Need Satisfaction scale (Van den Broeck et al., 2016), validated in Chinese organizational contexts (Xu et al., 2021). An item includes: “I have the freedom to decide what I do on my job.”

Psychological Distress was measured with the 7-item stress subscale from the Depression Anxiety Stress Scales-21 (DASS-21, Lovibond and Lovibond, 1995), which has demonstrated reliability across cultures (Wang et al., 2016). Participants indicated how often they experienced states like “feeling anxious.”

Psychological wellbeing was assessed using the 8-item Flourishing Scale (Diener et al., 2010), a concise measure of positive psychological functioning (e.g., “I lead a purposeful and meaningful life”).

Employee Resilience was measured with the 5-item Brief Resilience Scale (Smith et al., 2008), chosen for its focus on the core ability to bounce back (e.g., “I tend to bounce back quickly after hard times”).

The full items’ detail is given in Supplementary Appendix 1.

### 3.3 Data analysis strategy

The analysis followed a sequential, confirmatory approach using SPSS 28.0 and AMOS 28.0. First, descriptive statistics and bivariate correlations were examined. Second, Confirmatory Factor Analysis (CFA) was conducted to evaluate the discriminant validity and fit of the hypothesized six-factor measurement model. Third, the structural model was tested using covariance-based Structural Equation Modeling (SEM) to examine the direct and mediated pathways. The robust maximum likelihood estimator was used to account for potential deviations from normality.

To test the mediation hypotheses (H5, H6), we employed a bootstrapping procedure with 5,000 resamples to generate bias-corrected 95% confidence intervals for the indirect effects—a method preferred for its power and accuracy over traditional causal steps (Hayes, 2022). The moderating effects of resilience (H7a–H7d) were tested by creating latent interaction terms following the orthogonalizing approach outlined by Little et al. (2006), which reduces multicollinearity. Significant interactions were probed using simple slope analysis and visualized.

### 3.4 Addressing common method bias

Given the single-source design, we implemented both procedural and statistical remedies. Procedurally, we ensured anonymity, used clear and distinct scale anchors, and, crucially, employed temporal separation in measurement (Podsakoff et al., 2012, 2003). Statistically, we conducted two *post-hoc* tests. Harman’s single-factor test revealed the first factor explained only 18.0% of the variance, well below the 50% threshold. More definitively, we employed the marker variable technique, introducing a theoretically unrelated construct (general attitude toward technology) into the CFA model (Rönkkö and Ylitalo, 2011). The pattern and significance of the structural paths remained unchanged after partialling out its influence, indicating that common method variance is unlikely to confound the reported relationships.

## 4 Results

### 4.1 Preliminary analyses and measurement model

Prior to hypothesis testing, we evaluated the psychometric properties of our measures. Confirmatory factor analysis (CFA) supported the hypothesized six-factor structure (AI-based surveillance, AI-awareness, perceived autonomy, psychological distress, psychological wellbeing, employee resilience). The model demonstrated excellent fit to the data (χ^2^/df = 1.556, CFI = 0.978, TLI = 0.975, RMSEA = 0.034), exceeding conventional thresholds (Hair et al., 2017). As detailed in Table 2, all standardized factor loadings were significant and exceeded 0.70. Composite reliability (CR) values ranged from 0.845 to 0.937, and average variance extracted (AVE) for each construct surpassed 0.50, confirming strong internal consistency and convergent validity.

**TABLE 2:** Results of confirmatory factor analysis (CFA) and reliability assessment.

Constructs	Items	Factor loadings	Composite reliability	Average variance extracted
AI-based surveillance	AIS1	0.809	0.875	0.699
	AIS2	0.866		
	AIS3	0.833		
AI awareness	AIA1	0.849	0.903	0.699
	AIA2	0.853		
	AIA3	0.859		
	AIA4	0.781		
Perceived autonomy	PA1	0.771	0.845	0.645
	PA2	0.792		
	PA3	0.845		
Psychological distress	PD1	0.822	0.91	0.671
	PD2	0.867		
	PD3	0.735		
	PD4	0.867		
	PD5	0.798		
Psychological wellbeing	PWB1	0.774	0.937	0.651
	PWB2	0.824		
	PWB3	0.791		
	PWB4	0.796		
	PWB5	0.809		
	PWB6	0.789		
	PWB7	0.812		
	PWB8	0.859		
Employee resilience	ER1	0.79	0.876	0.586
	ER2	0.761		
	ER3	0.782		
	ER4	0.716		
	ER5	0.776		

PD6 and PD7 were deleted due to low factor loadings.

Discriminant validity was established using the Fornell-Larcker criterion (Fornell and Larcker, 1981). Table 3 shows the square root of each construct’s AVE (on the diagonal) was greater than its highest correlation with any other construct. Furthermore, correlations among the latent constructs were below 0.70, indicating that multicollinearity was not a concern for subsequent analysis (Johnson and LeBreton, 2004).

**TABLE 3:** Discriminant validity: correlations and square roots of AVEs.

Construct	CR	AVE	M	SD	α	1	2	3	4	5	6
1. Perceived autonomy	0.845	0.645	3.44	0.79	0.832	0.803	0.836	0.819	0.765	0.807	0.836
2. AI-based surveillance	0.875	0.699	3.41	0.82	0.861	–0.405					
3. Psychological distress	0.910	0.671	2.87	0.91	0.897	–0.596	0.306				
4. Employee resilience	0.876	0.586	3.28	0.76	0.858	0.042	–0.082	–0.238			
5. Psychological wellbeing	0.937	0.651	3.61	0.68	0.924	0.446	–0.312	–0.510	0.161		
6. AI-awareness	0.903	0.699	3.19	0.74	0.889	0.130	-0.042	-0.150	0.015	0.087	

Diagonal elements (in bold) are the square roots of the AVEs.

### 4.2 Hypothesis testing: direct and mediating effects

Structural equation modeling (SEM) was employed to test the hypothesized relationships. The structural model exhibited a good fit (χ^2^/df = 2.101, CFI = 0.962, TLI = 0.956, RMSEA = 0.048). The direct path coefficients, summarized in Table 4, provided strong support for H1 through H6.

**TABLE 4:** Hypothesis testing: structural model and direct effects.

Hypothesized path	b	β	SE	CR	*p*	Result
Perceived autonomy ← AI-awareness	0.111	0.093*	0.045	2.460	0.014	Supported
Psych. distress ← AI-awareness	–0.136	–0.117**	0.046	–2.958	0.003	Supported
Psych. distress ← AI-based surveillance	0.286	0.257***	0.043	6.614	< 0.001	Supported
Perceived autonomy ← AI-based surveillance	–0.359	–0.373***	0.044	–8.091	< 0.001	Supported
Psychological wellbeing ← perceived autonomy	0.250	0.290***	0.049	5.121	< 0.001	Supported
Psychological wellbeing ← psych. distress	–0.397	–0.531***	0.048	–8.267	< 0.001	Supported

b, unstandardized coefficient; β, standardized coefficient. SE, standard error; CR, critical ratio. *p < 0.05, **p < 0.01, ***p < 0.001. Effect sizes: | β| < 0.10 = negligible; 0.10–0.29 = small; 0.30–0.49 = medium; ≥ 0.50 = large (Cohen, 1992).

AI-based surveillance significantly undermined perceived autonomy (β = –0.359, *p* < 0.001) and heightened psychological distress (β = 0.286, *p* < 0.001). In contrast, AI-awareness fostered greater autonomy (β = 0.111, *p* = 0.014) and reduced distress (β = –0.136, *p* = 0.003). As expected, perceived autonomy was a positive predictor (β = 0.250, *p* < 0.001), and psychological distress a negative predictor (β = –0.397, *p* < 0.001), of psychological wellbeing.

The standardized coefficients show medium to large core structural path effects in terms of practical significance. The most significant effect in the model was the relationship between psychological distress and wellbeing (β = –0.531), which showed that a one standard deviation increase in distress would cause an increase in the psychological wellbeing by more than a half standard deviation, which is far more significant than traditional standards of organizational impacts (Cohen, 1992). The impact that AI-based surveillance has on perceived autonomy (β = –0.373) and distress (β = 0.257) is both medium-range, which means that surveillance is not just a statistically significant nuisance, but a practically significant cause of psychological damage.

To examine the proposed parallel mediation, a bootstrapping analysis with 5,000 resamples was conducted. The results, presented in Table 5, confirmed significant indirect effects for both AI-based surveillance and AI-awareness through the two mediators, thereby supporting the dual-pathway model.

**TABLE 5:** Bootstrapping analysis for indirect effects.

Indirect path	Point Est.	Boot SE	95% CI	Sig.	Total effect	% Mediated
AIS → perceived Autonomy → PWB	–0.090	0.020	[–0.132, –0.053]	Yes	–0.241	37.3%
AIS → psychological distress → PWB	–0.114	0.021	[–0.158, –0.075]	Yes	–0.241	47.3%
AIA → perceived autonomy → PWB	0.028	0.012	[0.006, 0.055]	Yes	0.093	30.1%
AIA → psychological distress → PWB	0.054	0.019	[0.020, 0.093]	Yes	0.093	58.1%

AIS, AI-Based Surveillance; AIA, AI-Awareness; PWB, Psychological wellbeing. All confidence intervals exclude zero. Total effect = sum of direct and all indirect effects. % Mediated = indirect effect ÷ total effect × 100. Bootstrap resamples = 5,000. Values are bias-corrected 95% CIs.

### 4.3 The moderating role of employee resilience

The analysis of moderating effects yielded findings that contradicted our initial buffering hypothesis for resilience. The interaction terms were tested within the SEM framework. Table 6 presents the key results for the moderation analysis.

**TABLE 6:** Results of moderating effect analysis.

Path (outcome: psychological distress)	b	β	SE	CR	*p*
AI-based surveillance (AIS)	0.272	0.244***	0.042	6.475	< 0.001
AI-awareness (AIA)	–0.136	–0.117**	0.042	–3.257	0.001
Employee resilience (ER)	–0.224	–0.188***	0.042	–5.267	< 0.001
INT1: AIS × ER [H7b—favored]	0.133	0.119***	0.035	3.794	<0.001
INT2: AIA × ER [H7d—favored]	0.094	0.077*	0.039	2.377	0.017
Path (outcome: perceived autonomy)	b	β	SE	CR	p
AI-based surveillance (AIS)	–0.356	–0.370***	0.043	–8.324	< 0.001
AI-awareness (AIA)	0.105	0.088*	0.043	2.474	0.013
Employee resilience (ER)	0.019	0.018 ns	0.043	0.444	0.657
INT1: AIS × ER [H7a—not supported]	–0.048	–0.041 ns	0.036	–1.355	0.175
INT2: AIA × ER [H7c—not supported]	0.007	0.006 ns	0.040	0.181	0.856

b, unstandardized; β, standardized coefficient. Green rows = significant interaction effects (theoretically favored direction). ns, non-significant. *p < 0.05, **p < 0.01, *** *p* < 0.001.

Contrary to H7a and H7c, resilience did not significantly moderate the relationships between the AI factors and perceived autonomy. The significant interactions emerged for psychological distress, but in the opposite direction to predictions. The positive relationship between AI-based surveillance and distress was stronger for employees with high resilience (β for interaction = 0.133, *p* < 0.001; H7b not supported). Similarly, the negative relationship between AI-awareness and distress was weaker for those high in resilience (β for interaction = 0.094, *p* = 0.017; H7d not supported). This pattern suggests resilience, rather than universally buffering stress, may under certain conditions amplify sensitivity to monitoring-related threats while muting the soothing effect of transparency—a dynamic aligning with recent critiques of resilience as a context-dependent construct (Britt et al., 2016).

Simple slope analysis revealed that among employees high in resilience (one SD above the mean: M + 0.76 = 4.04), a one-unit increase in AI-based surveillance predicted a 0.405-unit increase in psychological distress [b = 0.272 + 0.133 = 0.405, 95% CI (0.28, 0.53)]. Among employees low in resilience (one SD below the mean: M - 0.76 = 2.52), the same unit increase in surveillance predicted a substantially smaller 0.139-unit increase in distress [b = 0.272-0.133 = 0.139, 95% CI (0.04, 0.24)].

**FIGURE 2:**
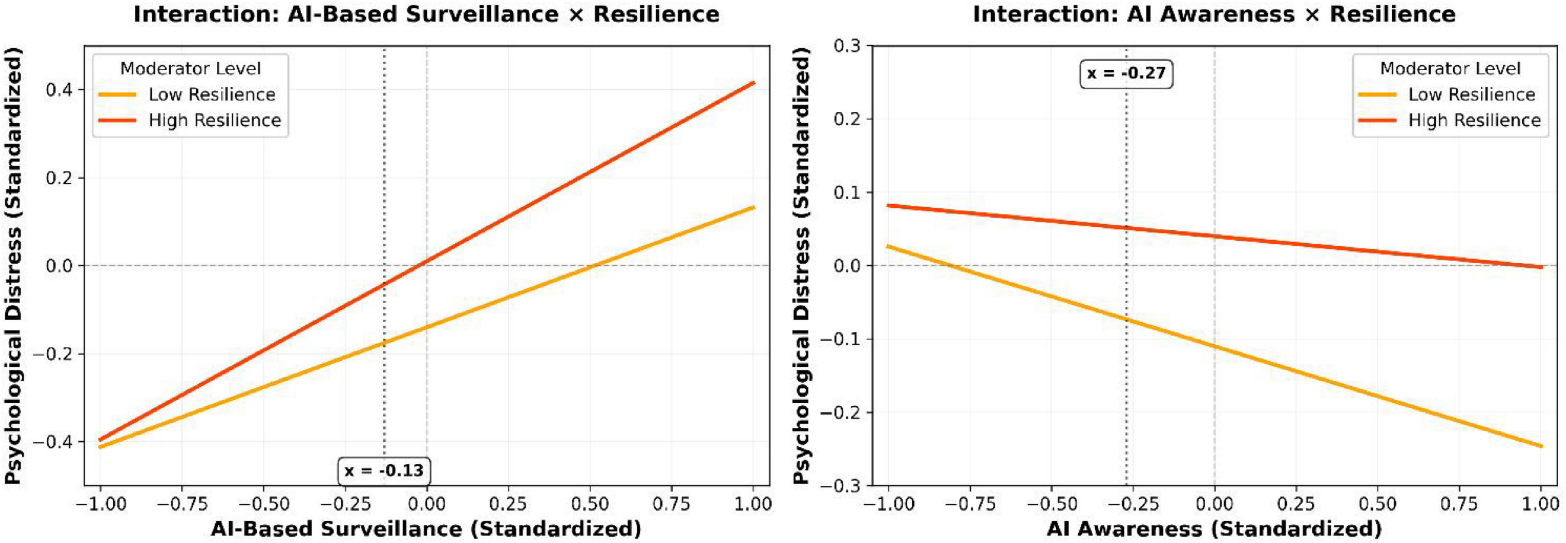
The moderating role of employee resilience on the relationships between AI factors and psychological distress.

Figure 2 illustrates these counterintuitive moderating effects through simple slopes plots. For high-resilience individuals, the slope between AI-based surveillance and distress is steeper, indicating a more pronounced negative reaction. Concurrently, the beneficial slope linking AI-awareness to reduced distress is flatter, implying their distress levels are less alleviated by awareness compared to their low-resilience peers.

### 4.4 Analysis of indirect effects

To more rigorously test the proposed mediation hypotheses (i.e., that AI factors influence psychological wellbeing through autonomy and distress), we conducted a bootstrapping analysis with 5,000 samples. The results, presented in Table 5, confirm the significance of the indirect effects. The indirect effect of AI-based surveillance on psychological wellbeing via perceived autonomy was significant [β = –0.090, 95% CI (–0.132, –0.053)], as was the indirect effect via psychological distress [β = –0.114, 95% CI (–0.158, –0.075)]. Similarly, the indirect effects of AI-awareness on psychological wellbeing through autonomy [β = 0.028, 95% CI (0.006, 0.055)] and through distress [β = 0.054, 95% CI (0.020, 0.093)] were also significant. These findings provide strong support for the proposed parallel mediation model.

## 5 Discussion

The widespread adoption of AI in the organizational life, especially in the competitive environment of the Chinese manufacturing industry, poses a multifaceted psychological environment of the employee. This paper aimed to chart this landscape by tracing the divergent directions in which AI-based surveillance and AI-awareness can affect managerial psychological wellbeing. Based on the S-O-R model, our model assumed perceived autonomy and psychological distress as parallel mediators and employee resilience as a major moderator. The results are mostly supportive of the hypothesized dual mediation directions and provide a more complex, and even provocative, contribution of resilience than traditional wisdom would assume.

### 5.1 Decoding the dual pathways

The findings strongly confirm the main idea that the psychological influence of AI is not universal but is essentially determined by the way it is manifested. The use of AI-based surveillance became a major danger to the psychological wellbeing, as it functions by undermining the sense of autonomy and intensifying mental distress. This is entirely in line with an emerging literature that criticizes algorithmic management. The data-driven surveillance that is inherent in such systems is continuous, which deprives employees of discretion and turns them into implementers of opaque measures (Cram et al., 2022). Our results support the opinion that this experience is not only inconvenient but also psychologically harmful because it causes hyper-vigilance and feelings of helplessness (Mettler, 2024). The large negative relationship between surveillance and autonomy (H1) and the positive relationship between surveillance and distress (H2) highlights that algorithmic control is a direct frustration to a fundamental psychological need, and at the same time, triggers a strong stress reaction.

On the other hand, AI-awareness was an effective counterpart that increased psychological wellbeing by promoting autonomy and reducing distress. This discovery is a strong argument in support of the values of openness and accountability of AI implementation. Once employees see the reason and manner in which AI tools work, the technology will no longer be an unintelligible menace, but an understandable and, possibly, empowering part of their workplace (Shin, 2021). The positive relationship with autonomy (H3) indicates that knowledge reinstates a feeling of agency, enabling employees to engage with AI in a strategically but not a reactive manner. The alleviation of distress (H4) also suggests that clarity alleviates the anxiety due to the uncertainty about employment and unfair appraisal, which is especially relevant when it comes to the fast automation of industries (Tong et al., 2021). These two cognitive-affective mechanisms are supported by the significant mediation effects via autonomy and distress (see Table 5) and give a more detailed picture than models with direct effects only.

### 5.2 The two-sided sword of resilience

The most interesting and theoretically important results relate to the moderating effect of employee resilience. In contrast to what our hypotheses (H7a–d) were founded on, its conventional conceptualization as a universal buffer, resilience proved to have a complex, context-specific effect, not a shield, but in fact, an amplifier of pressure, under some circumstances.

In particular, resilience reinforced the positive correlation between AI-surveillance and psychological distress (as opposed to H7b) and undermined the negative correlation between AI-awareness and distress (as opposed to H7d). The trend counters the mainstream discourse of positive organizational psychology that perceives resilience as an undisputed resource. Rather, it implies a pressure amplification effect in high stakes, monitored settings. The most resilient individuals, who often have a high level of achievement striving and a strong sense of individual responsibility (Lin and Yan, 2023), might not necessarily perceive AI-based surveillance as the threat that can be easily avoided but as a challenge that should be overcome. Such an increased involvement in the performance metrics dictated by the system may result in an increased cognitive and emotional involvement in the tasks being monitored, which is counterproductive and increases their psychological load (Kalischko and Riedl, 2024). They can internalize the system objectives, and this will result into self-pressure which increases distress- a situation that has been noticed in other high demand performance situations.

The given counterintuitive trend can also be explained with references to the challenge-hindrance appraisal model (Cavanaugh et al., 2000) and the recent critical approaches to resilience (Britt et al., 2016). The most resilient individuals, who are achievement-oriented, might be inclined to perceive AI-based surveillance not as an entirely negative hindrance, i.e., as a challenge, that needs to be overcome. Although such a challenge appraisal may be an effective way to generate engagement, it requires a long-term investment of cognitive and emotional resources, which results in the depletion of resources and increased distress. At the same time, their strong internal coping repertoires can also produce a ceiling effect, reducing the marginal utility of external resources such as AI-awareness in reducing stress. Conversely, weaker employees gain a lot of protection through the predictability and clarity of the awareness.

Equally, the reduced utility of AI-awareness to resilient employees implies that their already established, highly-developed coping repertoires can reduce the importance of external informational support. They may also turn to internal psychological resources to cope with stress more, which makes the effect of transparency less anxiety-reducing. In less resilient employees, however, AI-awareness seems to be an important external scaffold, the source of clarity and predictability that the internal resources of such employees cannot create on their own. This observation complicates the notion of a universal AI transparency program, meaning it has different psychological utility of different people.

It is important to put this finding into context in the context of our study: the manufacturing industry of China, where cultural values are based on hard work, hierarchy, and harmony among people (Xia and Liu, 2025). The pressure amplification effect on the resilient managers may be magnified in such an environment. Cultural norms to achieve the targets set by the authorities and preserve the status of the group might also stimulate their motivation to perform better, making AI-based surveillance a powerful source of performance anxiety instead of an indifferent instrument. This cultural layer introduces some boundary conditions to the generalizability of our model and recalls the demands of more culturally contextualized research on technology adoption (Farh et al., 2007).

### 5.3 Theoretical contributions

This research contributes in a number of ways in theory. First, it contributes to the development of the S-O-R framework by modeling and testing two parallel mediation routes successfully, going beyond a single mechanistic perspective. Second, it addresses recent demands of a more nuanced conceptualization of personal resources by showing that resilience may produce counter-intuitive results (Lin and Yan, 2023). Our results recommend an interactionist viewpoint of traits and situations, in which the valence of a trait such as resilience depends on the needs of the technological environment. Third, the research emphasizes the role of national culture and industry situation in modifying basic psychological processes by studying the manufacturing of China, and recommends that future studies should not regard context as noise but as a theoretical variable.

In addition to the fact that this study helps to test the universal psychological processes that are captured in our model, it is important to note that contextualization of organizational theory is critical. The particular situation of the manufacturing industry in China, with its high Power Distance and collectivist norms (Hofstede Insights, 2023; Farh et al., 2007), is not only a sampling setting but also an effective culture booster of the dynamics observed. The perceived inescapability and legitimacy of top-down AI-based surveillance is probably heightened by high power distance and thus makes its erosion of perceived autonomy potentially worse. On the other hand, the experience of distress may be moderated by collectivist values, which place more emphasis on group harmony and performance, so that fears of not meeting expectations of the team in an algorithmic evaluation may exacerbate personal anxiety. Thus, we can propose that the magnitude of the hypothesized pathways, especially that of surveillance and its interaction with resilience, can be culture-specific. This leads to an important theoretical implication: technology adoption and employee psychological wellbeing should be modeled with the cultural dimensions as explicit moderators or a boundary condition, and that the future of theories in the context should be more specific and less general, that is, specifying when, who, and in what cultural contexts the effects are strengthened or weakened.

### 5.4 Practical contributions

The use of AI-based surveillance is not a neutral implementation choice, as it has significant negative effects on autonomy and increases distress, and these negative effects spread to psychological wellbeing in two different psychological mechanisms. HR departments cannot make deployment a technical process. Organizations ought to engage employees in defining data limits and limiting monitoring to task-relevant metrics before activation and create a mechanism of appealing against algorithmic decisions. The AI literacy training should be mandatory and not subsequent to exposure to monitoring since its protective effect is the best when employees are exposed to the surveillance environments with clarity on how the system operates. Continuous pulse survey and regular audits at the time offer the early warning mechanism required to identify the worsening and avert scope creep.

The level of resilience essentially redefines the risk profile of an employee under surveillance, and a homogenous support strategy is scientifically untenable as well as a waste of resources. Employees with high-resilience—who are generally assumed to require little support, are at high risk of amplification of pressure: the achievement-striving orientation makes them internalize algorithmic metrics as personal problems, producing self-imposed pressure that exacerbates system-generated distress. In this group, perfectionism-awareness coaching, psychological detachment training, and explicit manager briefing should be offered to the organization to identify and disrupt upward standard-setting. The use of AI-awareness training is not enough since transparency does not bring much relief to employees who already have strong internal coping mechanisms. The opposite setup applies to low-resilience employees: acutely reliant on clarity as external scaffold, they need to be walked through evaluation metrics individually, have a designated AI liaison contact, and gradual exposure protocol that increases monitoring intensity over time.

### 5.5 Limitations and future directions

Although it is a three-wave time-lagged design, it is inappropriate to make definite causal claims. Temporal separation provides precedence but does not exclude reverse causation—workers who already feel highly distressed might feel that surveillance is more intimidating, or lower-well workers might declare reduced autonomy despite the absence of actual monitoring. Experimental or quasi-experimental designs, including natural experiments that take advantage of staggered AI rollouts across organizational units, would be able to offer better causal leverage than survey methodology can offer by itself.

Despite the use of procedural and statistical remedies, all the constructs were self-reported by the same participants and this could have introduced the risk of shared response tendencies that may inflate observed associations to a degree that the CMV statistics cannot fully capture. The high power-distance manufacturing environment of China presents a unique challenge in terms of social desirability because employees might report less distress caused by surveillance or more autonomy to seem non-resistant. The next generation of research must include both supervisor rated or organizationally documented performance data and self-report measures in order to triangulate results.

There are a number of measurement decisions that should be mentioned. First, every scale was short (three to eight items) which, although pragmatically suitable in longitudinal surveys, compromises construct coverage. The autonomy subscale specifically represents a very limited portion of self-determination and might not be sensitive to more subtle forms of algorithmic constraint. Second, the measure of resilience was conducted at T3 and not T1. In spite of the theorized stability of resilience as a trait, the accumulation of resilience 6 months into the study is a risk of contamination by the same surveillance experiences under study, which may overstate its relationship with distress responses at the same time. The measurement of the resilience should be done at the baseline before any exposure measurement can be done. Third, AI-awareness and AI-based surveillance were assessed based on self-report perceptions and not objective system parameters, i.e., the results are related to psychological experience of monitoring and not its technical intensity, which is a significant interpretive boundary condition.

The sample is limited to the manufacturing industry managers in three provinces in China, which constrains generalizability on multiple dimensions at the same time. The manufacturing setting entails more production-metric-based AI monitoring than the knowledge-work or service setting; the managerial sample does not include frontline workers, who can be more directly and intensively monitored; and the collectivist, high power-distance cultural setting may increase both the pressure created by surveillance and norm-based coping in ways not generalizable to individualistic settings. Professional service, healthcare, or public sector contexts Replication is required before the model can be considered universal.

Latent interaction modeling through the orthogonalizing methodology has a number of assumptions that are worth consideration: it assumes that the latent variables are normally distributed, the effects of interaction are linear and that there is measurement invariance of the resilience levels. The linearity assumption is especially significant, as the effect of amplification of pressure can actually be threshold-dependent, and it can be strong only above a given level of surveillance, instead of being uniform throughout the entire range. This non-linearity could be directly tested with a future study by using a response surface analysis or a polynomial regression.

## Data Availability

The raw data supporting the conclusions of this article will be made available by the authors, without undue reservation.
