# Supplementary material for: When resilience backfires: the counterintuitive effect of employee resilience in high-tech surveillance environments
Source: Front Psychol. 2026 Mar 19;17:1790846. doi: 10.3389/fpsyg.2026.1790846 (PMC13043328; doi:10.3389/fpsyg.2026.1790846)
Supplement: Supplementary file 1 [file Table_1.docx]

**Appendix A**

**AI-Based Surveillance**

AI-based technologies are used to track my work activities and progress

My Performance is frequently evaluated through AI-based technologies

I am Rarely monitored at job through AI-based technologies

**AI-Awareness**

I think my job could be replaced by AI

I am personally worried that what I do now in my job will be able to be replaced by AI

I am personally worried about my future in my organisation due to AI replacing employees

I am personally worried about my future in my industry due to AI replacing employees

**Perceived Autonomy**

I have the freedom to decide what I do on my job

It is basically my own responsibility to decide how my job gets done

I have a lot of say about what happens on my job.

**Psychological Distress**

I found it hard to wind down

I tended to over-react to situations

I felt that I was using a lot of nervous energy

I found myself getting agitated

I found it difficult to relax

I felt that I was rather touchy

I was intolerant of anything that kept me from getting on with what I was doing

**Psychological Well-being**

I lead a purposeful and meaningful life

My social relationships are supportive and rewarding

I am engaged and interested in my daily activities

I actively contribute to the happiness and well-being of others

I am competent and capable in the activities that are important to me

I am a good person and live a good life

I am optimistic about my future

People respect me

**Employee Resilience**

I tend to bounce back quickly after hard times.

I have a hard time making it through stressful events (R)

It does not take me long to recover from a stressful event

It is hard for me to snap back when something bad happens (R)

I usually come through difficult times with little trouble

I tend to take a long time to get over set-backs in my life (R)
